# The phylogeographical pattern of the Amur minnow *Rhynchocypris lagowskii* (Cypriniformes: Cyprinidae) in the Qinling Mountains

**DOI:** 10.1002/ece3.8924

**Published:** 2022-05-15

**Authors:** Tao Chen, Li Jiao, Lili Ni

**Affiliations:** ^1^ 74716 Guangxi Key Laboratory of Diabetic Systems Medicine Guilin Medical University Guilin P.R. China; ^2^ 74716 Faculty of Basic Medical Sciences Guilin Medical University Guilin P.R. China; ^3^ 12401 College of Life Sciences Shaanxi Normal University Xi’an P.R. China

**Keywords:** *Cytb*, phylogeographical pattern, Qinling Mountains, *Rhynchocypris lagowskii*

## Abstract

In this study, the phylogeographical pattern of the Amur minnow (*Rhynchocypris lagowskii*) widely distributed in the cold freshwaters of the Qinling Mountains was examined. A total of 464 specimens from 48 localities were sequenced at a 540‐bp region of the mitochondrial cytochrome b (*Cytb*) gene, and 69 haplotypes were obtained. The mean ratio of the number of synonymous and nonsynonymous substitutions per site (dN/dS) was 0.028 and indicated purifying selection. Haplotype diversity (*h*) and nucleotide diversity (*π*) of natural populations of *R*. *lagowskii* varied widely between distinct localities. Phylogenetic trees based on Bayesian inference (BI), maximum likelihood (ML), and maximum parsimony (MP) methods, and network analysis showed five well‐differentiated lineages, but these did not completely correspond to localities and geographic distribution. Meanwhile, analysis of molecular variances (AMOVA) indicated the highest proportion of genetic variation was attributed to the differentiation between populations rather than by our defined lineages. In addition, there was no significant correlation between the pairwise Fst values and geographic distance (*p *> .05). Based on the molecular clock calibration, the time to the most recent common ancestor (TMRCA) was estimated to have emerged from the Late Miocene to the Early Pleistocene. Finally, the results of demographic history based on the neutrality test, mismatch distribution, and Bayesian skyline plot (BSP) analyses showed that collectively, the populations were stable during the Pleistocene while one lineage (lineage E) probably underwent a slight contraction during the Middle Pleistocene and a rapid expansion from the Middle to the Late Pleistocene. Therefore, the study suggests the current phylogeographical pattern of *R*. *lagowskii* was likely shaped by geological events that led to vicariance followed by dispersal and secondary contact, river capture, and climatic oscillation during the Late Miocene to the Early Pleistocene in the Qinling Mountains.

## Graphical abstract

The study suggests that the current phylogeographical pattern of R. lagowskii was likely shaped by geological events that led to the past vicariance followed by the dispersal and secondary contact, river capture, and climatic oscillation during the Late Miocene to the Early Pleistocene in the Qinling Mountains.

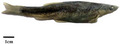

## 1 INTRODUCTION

Phylogeography is the study of historical processes that may be responsible for the contemporary geographic distributions of genealogical lineages within and among closely related species and is primarily conducted using molecular markers (Avise, 2000; Bowen et al., 2016; Chen et al., 2020; Hardouin et al., 2018; Li et al., 2011; Schneider et al., 1998; Wang et al., 2012; Wu et al., 2009; Yu et al., 2014). It can be used to identify different historical forces, such as population expansion, bottlenecks, climate oscillation, vicariance, and migration, analyze the variation in population distributions, and reconstruct the evolutionary processes of fauna and flora (Huang, 2012). Animal mitochondrial DNA (mtDNA) had been widely used in phylogenetics for systematic, population genetics, phylogeography, and comparative phylogeography (Avise et al., 1987; Bowen et al., 2016; Chen et al., 2020; Hardouin et al., 2018; Yu et al., 2014) and was employed in this study due to its maternal inheritance, rapid mutation rate, and low level of intermolecular genetic recombination (Brown et al., 1979; Clayton, 1982; Giles et al., 1980).

The Qinling Mountains represent a natural boundary between the northern and southern regions of the country and separate the Chinese temperate and subtropical climatic zones (Ding et al., 2013), resulting in differentiated terrestrial and freshwater fauna (Li, 1981; Zhang, 2011). Meanwhile, the rapid uplift of these mountains and climatic oscillation were influenced by the Qinghai–Tibet Plateau movement from the Miocene to the Pleistocene and have played important roles in influencing the phylogeographical patterns of a variety of organisms, including parasite, amphibian, fish, and mammal species (Chen et al., 2020; Hardouin et al., 2018; He et al., 1992; Hu et al., 2021; Huang et al., 2017; Li et al., 2021; Liu et al., 2015; Meng et al., 2014; Shao et al., 2019; Shi, 2002; Wang et al., 2012, 2013; Yu et al., 2014; Zhang & Fang, 2012).

**FIGURE 1:**
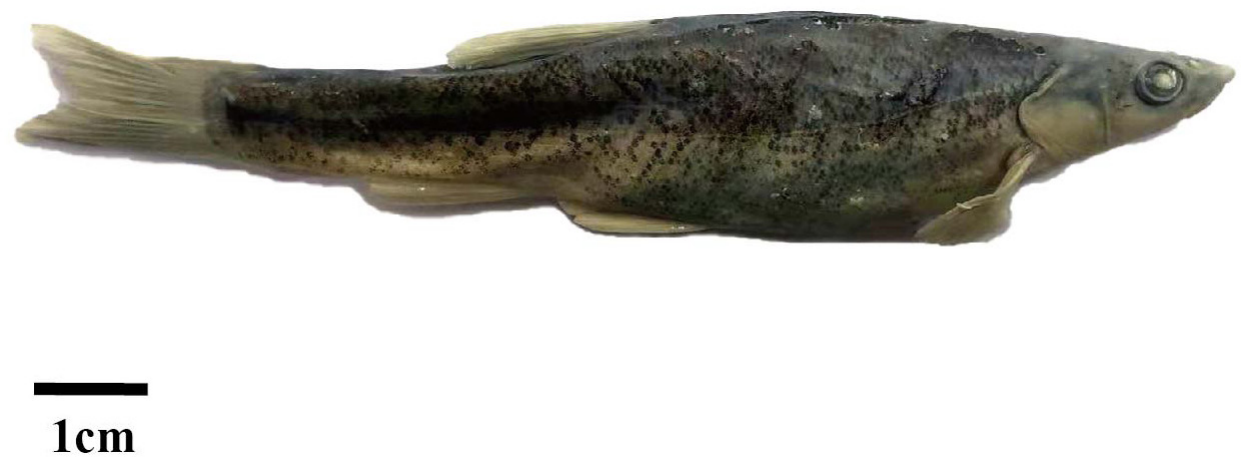
Amur minnow, *Rhynchocypris lagowskii*

The Amur minnow (*Rhynchocypris lagowskii*) (Figure 1) is a small cyprinid widely distributed in cold freshwater from the Lena and the Amur Rivers southward to the Yangtze drainage in East Asia (Bogutskaya et al., 2008; Chen, 1998; Min & Yang, 1986). *R*. *lagowskii* and related fish species were selected for phylogeographic studies because their specific ecological upstream distribution resulted in much smaller population sizes, low dispersal ability, and restricted gene flow (Hassan et al., 2015; Higuchi & Watanabe, 2005; Kang et al., 2000; Min & Yang, 1986; Nishida et al., 2015; Sakai et al., 2014; Xue et al., 2017; Yu et al., 2014; Zhang & Chen, 1997). The divergence times of other parasite and fish species in this region were estimated to have occurred during the Early to the Middle Pleistocene and followed the rapid uplift of the Qinling Mountains (Chen et al., 2020; Hardouin et al., 2018; Yu et al., 2014; Zhang & Fang, 2012). However, the time to the most recent common ancestor (TMRCA) of the congeneric species *Rhynchocypris oxycephalus*, *Rhynchocypris percnurus*, and *R*. *lagowaskii* was estimated to be in the Late Miocene. As a consequence, the phylogeographical pattern of *R*. *oxycephalus* was shaped by the geological events and Pliocene climate fluctuations, but this study only used four individuals of *R*. *lagowaskii* as the outgroup taxa (Yu et al., 2014). Unfortunately, there have been limited studies and insufficient sampling of *R*. *lagowskii* in the Qinling Mountains (Yu et al., 2014), so it has been difficult to evaluate the phylogeographical pattern of *R*. *lagowskii* in this region. Therefore, the purpose of the study is to use a larger number of specimens and cover a wide geographical range in the Qinling Mountains to illustrate the phylogeographical pattern of the species. For this work, we explored the phylogeographical pattern of *R*. *lagowskii*, based on the c ytochrome b (*Cytb*) gene sequences of 464 specimens from 48 geographical localities in the main Qinling Mountains. In addition, the study also assessed genetic differentiation between populations, demographic history, and the effects of geological events or climate oscillation during the Pleistocene.

## 2 MATERIALS AND METHODS

### 2.1 Ethics statement

The study was approved by the Animal Care and Use Committee of Shaanxi Normal University. The species is not evaluated in the IUCN red list status (https://www.iucnredlist.org). None of the species sampled are endangered or protected in China (Yue & Chen, 1998). Fish sampling is permitted by the local level authority in scientific research.

### 2.2 Sample collection

**FIGURE 2:**
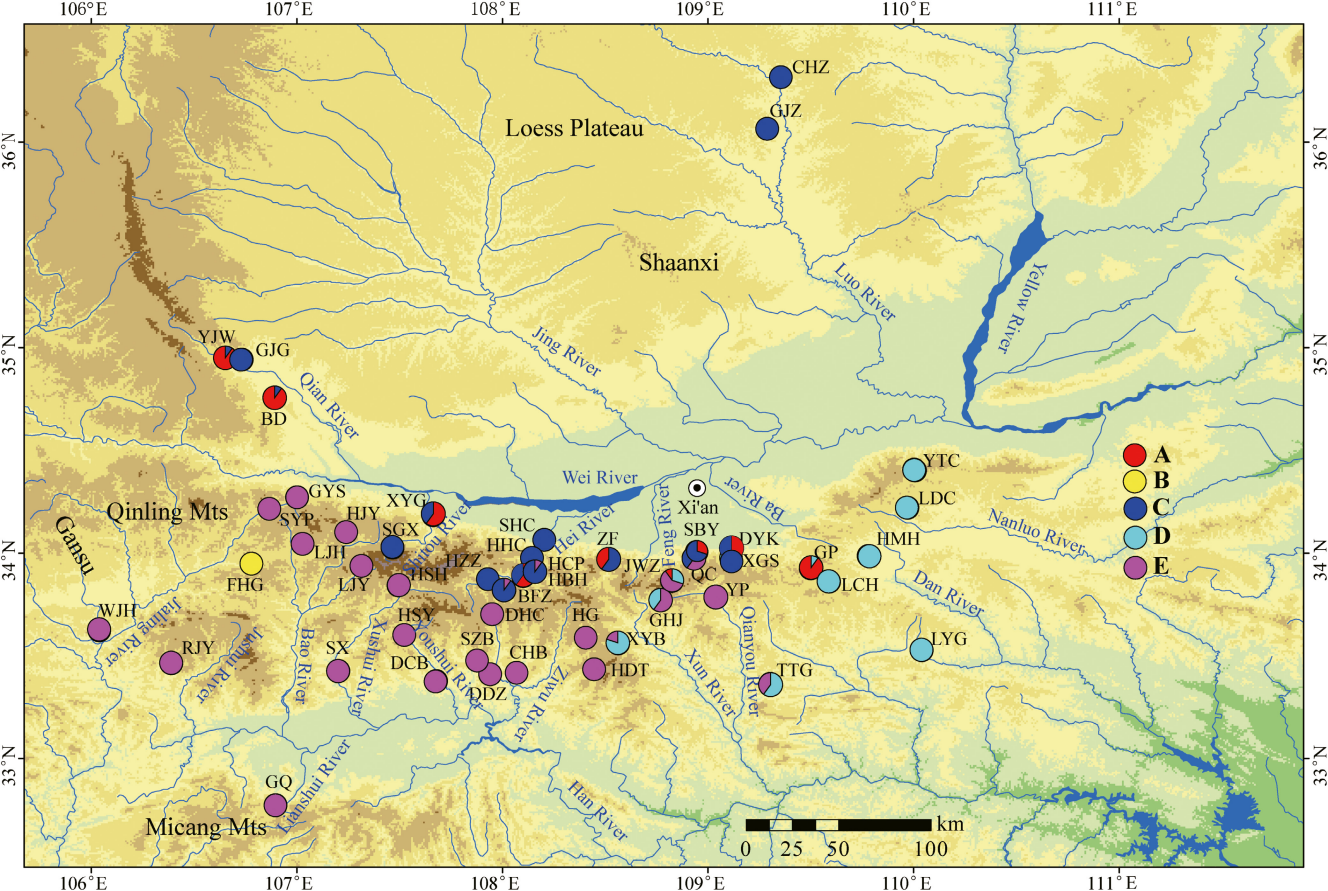
Map of sampling localities for *Rhynchocypris lagowskii* populations. The map was downloaded from the National Geomatics Center of China with slight modification using Arcgis10.1. The locality codes are given in Table 1. The populations belonged to different lineages labelled red (a), yellow (b), blue (c), cyan (d), and purple (e), respectively

Specimens of *R*. *lagowskii* were sampled from 48 localities, which covered most regions of the Qinling Mountains, from May to October in 2016 and 2017 (Figure 2 and Table 1). The fish were rapidly euthanized by a blow to the head and stored in 96% ethanol within three minutes. Subsequently, the specimens were examined microscopically in the laboratory, and species identification was performed based on the morphological characteristics (Chen, 1998). A total of 464 specimens were identified. Finally, specimens were individually stored in 96% ethanol at 4°C. Voucher specimens were deposited in the Fish Disease Laboratory, College of Life Sciences, Shaanxi Normal University, Xi’an, China, 710062 (Accession number: Acc.RL2017001‐2017464).

**TABLE 1:** Sampling information and haplotype diversity based on the *Cytb* sequences for 48 localities of *Rhynchocypris lagowski*

Population code	Locality	Lineage	n/N	Coordinates	Altitude (m)	Haplotypes	h	π
BD	Long Co., Shaanxi Prov.	A/C	7/10	N34.756858°/E106.890432°	926.1	Hap1(1), Hap2(1), Hap3(2), Hap4(2), Hap5(1), Hap6(1), Hap7(2)	0.933 ± 0.062	0.0262 ± 0.0154
BFZ	Zhouzhi Co., Shaanxi Prov.	C/E	3/10	N33.819712°/E108.010060°	1100.0	Hap8(8), Hap9(1), Hap10(1)	0.378 ± 0.181	0.0130 ± 0.00970
CHB	Foping Co., Shaanxi Prov.	E	1/10	N33.417045°/E108.069953°	665.4	Hap10(10)	**–**	**–**
CHZ	Ganquan Co., Shaanxi Prov.	C	1/10	N36.316173°/E109.354632°	1032.0	Hap11(10)	**–**	**–**
DCB	Yang Co., Shaanxi Prov.	E	1/1	N33.376617°/E107.679210°	623.8	Hap10(1)	**–**	**–**
DDZ	Foping Co., Shaanxi Prov.	E	1/10	N33.410476°/E107.940753°	964.5	Hap10(10)	**–**	**–**
DHC	Foping Co., Shaanxi Prov.	E	2/10	N33.702610°/E107.949857°	1466.9	Hap10(7), Hap12(3)	0.467 ± 0.132	0.000879 ± 0.00024
DYK	Chang’an Co., Shaanxi Prov.	A/C	3/10	N34.018042°/E109.115260°	720.7	Hap13(5), Hap14(2), Hap15(3)	0.689 ± 0.104	0.00570 ± 0.00748
FHG	Feng Co., Shaanxi Prov.	B	3/10	N33.941831°/E106.732453°	1110.4	Hap16(4), Hap17(2), Hap18(4)	0.711 ± 0.086	0.00264 ± 0.00041
GHJ	Ningshan Co., Shaanxi Prov.	D/E	3/10	N33.773644°/E108.769971°	1412.0	Hap19(4), Hap20(5), Hap21(1)	0.644 ± 0.101	0.0179 ± 0.00031
GJG	Long Co., Shaanxi Prov.	C	6/10	N34.951040°/E106.655067°	1112.5	Hap6(1), Hap22(4), Hap23(2), Hap24(1), Hap25(1), Hap26(1)	0.844 ± 0.103	0.00425 ± 0.00078
GJZ	Fu Co., Shaanxi Prov.	C	2/10	N36.064353°/E109.288433°	1035.2	Hap11(9), Hap27(1)	0.200 ± 0.154	0.000374 ± 0.000293
GP	Lantian Co., Shaanxi Prov.	A/D	3/10	N33.925229°/E109.499600°	1049.4	Hap28(7), Hap29(2), Hap30(1)	0.511 ± 0.164	0.0202 ± 0.0145
GQ	Nanzheng Co., Shaanxi Prov.	E	1/10	N32.773223°/E106.896475°	1001.3	Hap31(10)	**–**	**–**
GYS	Weibin Co., Shaanxi Prov.	E	1/10	N34.271956°/E107.001503°	892.0	Hap32(10)	**–**	**–**
HBH	Zhouzhi Co., Shaanxi Prov.	A/C	4/10	N33.887652°/E108.101550°	788.3	Hap8(3), Hap14(3), Hap33(1), Hap34(3)	0.800 ± 0.076	0.0552 ± 0.00961
HCP	Zhouzhi Co., Shaanxi Prov.	C/E	2/10	N33.910060°/E108.152578°	1028.7	Hap8(9), Hap10(1)	0.200 ± 0.154	0.0126 ± 0.00972
HDT	Ningshan Co., Shaanxi Prov.	E	2/10	N33.434294°/E108.445810°	1529.1	Hap10(8), Hap21(2)	0.356 ± 0.159	0.00595 ± 0.00267
HG	Ningshan Co., Shaanxi Prov.	E	2/10	N33.587632°/E108.406290°	1315.5	Hap10(3), Hap35(7)	0.467 ± 0.132	0.000879 ± 0.00024
HHC	Zhouzhi Co., Shaanxi Prov.	C	2/10	N33.976385°/E108.144350°	780.5	Hap8(3), Hap33(7)	0.467 ± 0.132	0.000879 ± 0.00024
HJY	Taibai Co., Shaanxi Prov.	E	5/10	N34.101574°/E107.240058°	1669.4	Hap10(5), Hap32(1), Hap36(1), Hap37(2), Hap38(1)	0.756 ± 0.130	0.001778 ± 0.00045
HMH	Shangzhou Co., Shaanxi Prov.	D	3/10	N33.986237°/E109.781073°	843.4	Hap39(5), Hap40(3), Hap41(2)	0.689 ± 0.104	0.00153 ± 0.00033
HSH	Taibai Co., Shaanxi Prov.	E	4/10	N33.842181°/E107.494487°	1390.3	Hap42(6), Hap43(2), Hap44(1), Hap45(1)	0.644 ± 0.152	0.00421 ± 0.00112
HSY	Yang Co., Shaanxi Prov.	E	2/10	N33.601236°/E107.522693°	1171.3	Hap10(8), Hap46(2)	0.356 ± 0.159	0.000667 ± 0.00030
HZZ	Zhouzhi Co., Shaanxi Prov.	C	2/10	N33.872799°/E107.929752°	1087.5	Hap8(9), Hap33(1)	0.200 ± 0.154	0.000374 ± 0.000293
JWZ	Chang’an Co., Shaanxi Prov.	A/D/E	3/10	N33.864368°/E108.824842°	1685.1	Hap19(3), Hap21(6), Hap47(1)	0.600 ± 0.131	0.0319 ± 0.0129
LCH	Shangzhou Co., Shaanxi Prov.	D	2/10	N33.861331°/E109.589170°	1074.1	Hap39(2), Hap41(8)	0.356 ± 0.159	0.000667 ± 0.00030
LDC	Huazhou Co., Shaanxi Prov.	D	2/3	N34.219980°/E109.972002°	1130.7	Hap48(2), Hap49(1)	0.667 ± 0.314	0.0124 ± 0.000586
LJH	Feng Co., Shaanxi Prov.	E	2/10	N34.045245°/E107.027867°	1381.6	Hap10(7), Hap50(3)	0.467 ± 0.132	0.00173 ± 0.000495
LJY	Taibai Co., Shaanxi Prov.	E	3/10	N33.937146°/E107.314023°	1637.4	Hap10(6), Hap37(3), Hap51(1)	0.600 ± 0.131	0.00136 ± 0.000384
LYG	Shanyang Co., Shaanxi Prov.	D	2/10	N33.526551°/E110.039283°	830.7	Hap39(9), Hap40(1)	0.200 ± 0.154	0.000374 ± 0.000293
QC	Chang’an Co., Shaanxi Prov.	C/E	2/10	N33.977764°/E108.932698°	794.6	Hap13(4), Hap20(6)	0.533 ± 0.095	0.0307 ± 0.00544
RJY	lueyang Co., Shaanxi Prov.	E	1/10	N33.463460°/E106.389318°	1070.3	Hap52(10)	**–**	**–**
SBY	Chang’an Co., Shaanxi Prov.	A/C	3/10	N34.005723°/E108.946291°	625.4	Hap13(7), Hap14(1), Hap53(2)	0.511 ± 0.164	0.0469 ± 0.0133
SGX	Taibai Co., Shaanxi Prov.	C	6/10	N34.026913°/E107.466723°	1500.7	Hap13(5), Hap54(1), Hap55(1), Hap56(1), Hap57(1), Hap58(1)	0.778 ± 0.137	0.00421 ± 0.00125
SHC	Zhouzhi Co., Shaanxi Prov.	C	4/10	N34.063913°/E108.204107°	493.3	Hap8(3), Hap13(4), Hap59(2), Hap60(1)	0.778 ± 0.091	0.00190 ± 0.000374
SX	Chenggu Co., Shaanxi Prov.	E	2/10	N33.426384°/E107.203005°	658.0	Hap43(7), Hap61(3)	0.467 ± 0.132	0.00433 ± 0.00122
SYP	Feng Co., Shaanxi Prov.	E	1/10	N34.215707°/E106.865443°	1386.3	Hap10(10)	**–**	**–**
SZB	Yang Co., Shaanxi Prov.	E	2/10	N33.477182°/E107.877315°	845.7	Hap10(9), Hap62(1)	0.200 ± 0.154	0.000374 ± 0.000293
TTG	Zhen’an Co., Shaanxi Prov.	D/E	3/10	N33.363943°/E109.304154°	1053.1	Hap20(2), Hap21(2), Hap39(6)	0.622 ± 0.138	0.0175 ± 0.00313
WJH	Hui Co., Gansu Prov.	E	1/10	N33.622921°/E106.040017°	732.4	Hap63(10)	**–**	**–**
XGS	Chang’an Co., Shaanxi Prov.	C	1/10	N33.976884°/E109.112943°	1110.6	Hap13(10)	**–**	**–**
XYB	Ningshan Co., Shaanxi Prov.	D/E	3/10	N33.559413°/E108.563282°	1384.3	Hap19(3), Hap64(5), Hap65(2)	0.689 ± 0.104	0.0122 ± 0.00503
XYG	Mei Co., Shaanxi Prov.	A/C	4/10	N34.183966°/E107.663465°	677.4	Hap4(1), Hap7(2), Hap29(3), Hap60(4)	0.778 ± 0.091	0.0572 ± 0.00964
YJW	Long Co., Shaanxi Prov.	A/C	3/10	N34.959532°/E106.790017°	1006.1	Hap6(1), Hap7(8), Hap15(1)	0.378 ± 0.181	0.0228 ± 0.0163
YP	Zhashui Co., Shaanxi Prov.	E	2/10	N33.784181°/E109.035646°	1106.2	Hap20(7), Hap21(3)	0.467 ± 0.132	0.000879 ± 0.00024
YTC	Huayin Co., Shaanxi Prov.	D	3/10	N34.401592°/E110.007253°	1440.1	Hap48(8), Hap66(1), Hap67(1)	0.378 ± 0.181	0.00111 ± 0.00061
ZF	Huyi Co., Shaanxi Prov.	A/C	4/10	N33.967430°/E108.518078°	659.1	Hap4(3), Hap59(5), Hap68(1), Hap69(1)	0.711 ± 0.117	0.0552 ± 0.00960
Total	48 localities	A‐E	69/464	N32.773223°‐N36.316173° /E106.040017°‐E110.039283°	623.8–1669.4	Hap1(1)‐Hap69(1)	0.942 ± 0.006	0.0514 ± 0.00188

Note

Abbreviations in title: n, the number of *Cytb* haplotypes; N, the number of individuals; h, haplotype diversity; π, nucleotide diversity.

### 2.3 DNA extraction, PCR amplification, and direct sequencing

Total genomic DNA of *R*. *lagowskii* was extracted from each specimen following the operation instruction of the TIANamp Marine Animals DNA Kit (Tiangen Biotech, Beijing, China). The forward primer Cytb‐F (5’‐ATGGCAAGCCTACGAAAAAC‐3’) and the reverse primer Cytb‐R (5’‐GATTACAAGACCGATGCTTT‐3’) designed based on the same species (Zhao et al., 2016) were used to amplify a 540‐bp fragment of the mitochondrial c ytochrome b (*Cytb*) gene by polymerase chain reaction (PCR) for each specimen. PCR amplification was performed in a total volume of 25 µL, containing 3 mM MgCl_2_, 10 mM Tris‐HCl (pH 8.3), 50 mM KCl, 0.25 mM of each dNTP, 1.25 U rTaq polymerase (TaKaRa, Dalian, China), 0.4 μM of each primer, 45 ng gDNA, tapped with Milli‐Q water. The following cycling conditions were applied: initial denaturation for 1 min at 93°C followed by 35 cycles of denaturation for 10 s at 92°C, annealing for 1.5 min at 51°C, and extension for 2 min at 60°C with a final extension for 6 min at 72°C (Chen et al., 2020). All fragments were initially purified with a PCR purification kit (BGI Biotech, Shenzhen, China), subsequently subjected to electrophoresis in a 1% agarose gel, and finally sequenced with the forward primer using an ABI Prism®3730 automated sequencer (Applied Biosystems, Foster City, USA).

### 2.4 Data analyses

#### 2.4.1 Population genetic diversity

A total of 464 *Cytb* gene sequences were visually inspected and manually edited using BioEdit v7.0.9.0 (Hall, 1999) and then aligned with MUSCLE (Edgar, 2004) as implemented in MEGA v6.06 (Tamura et al., 2013). Subsequently, the ratio of nonsynonymous and synonymous substitutions per site (dN/dS) using maximum likelihood (ML) analysis of natural selection codon‐by‐codon via HyPhy and nucleotide composition were calculated with MEGA v6.06 (Tamura et al., 2013). In addition, the genetic diversity indices for the number of haplotypes (*n*), haplotype diversity (*h*), and nucleotide diversity (*π*) were calculated using DnaSP v5.10.1 (Librado & Rozas, 2009). Finally, all haplotype sequences were deposited in GenBank under accession numbers MW831313‐MW831381.

#### 2.4.2 Phylogenetic and network analyses

Before phylogenetic analysis, a substitution model for the haplotype dataset was determined using the Bayesian information criterion (BIC) in jModelTest v2.2.10 (Darriba et al., 2012). As a result, the TrN model (Tamura & Nei, 1993) of molecular evolution with the gamma shape parameter (TrN+G) was selected. Subsequently, phylogenetic trees based on the mitochondrial *Cytb* haplotypes were reconstructed using the Bayesian inference (BI), maximum likelihood (ML), and maximum parsimony (MP) methods. The congeneric species *R*. *percnurus* was selected as an outgroup (Imoto et al., 2013). Maximum parsimony analysis was implemented in PAUP* v4.0b10a (Swofford, 2002). Heuristic searches with tree‐bisection‐reconnection were executed for 1000 random addition replicates with all characters treated as unordered and equally weighted. Maximum likelihood analysis was conducted using RAxML v7.2.8 (Stamatakis et al., 2005), with bootstrap analysis performed with 1,000 replicates. Bayesian inference analysis was performed using MrBayes v3.1.2 (Ronquist & Huelsenbeck, 2003), and one set of four chains was allowed to run simultaneously for 15 million generations. The trees were sampled every 1000 generations, with the first 25% being discarded as burn‐in. Stationarity means that the log‐likelihood kept a stable level with the sampled generations increasing, and it was considered to be reached when the average standard deviation of split frequencies was below 0.01. A median‐joining haplotype network was then constructed using PopART v1.7 (Leigh & Bryant, 2015).

#### 2.4.3 Population genetic structure

The mean genetic distances among the lineages identified in our phylogenetics analysis (see results) were calculated by an uncorrected p‐distance model using MEGA v6.06 (Tamura et al., 2013). Subsequently, the analysis of molecular variances (AMOVA) was performed to investigate the level of genetic variation between populations using pairwise differences F‐statistics in Arlequin v3.5.1.2 (Excoffier & Lischer, 2010). Finally, the correlation between the pairwise Fst values of the individuals from localities and their geographic distances (km) was analyzed to test for isolation by distance (IBD) (Slatkin, 1993) and assessed using linear regression in GraphPad Prism v 5.0 (www.graphpad.com).

#### 2.4.4 Divergence time estimation

For divergence time estimation among the lineages identified from the well‐supported phylogenetic clades, a coalescent time estimation method was used in BEAST v1.6.1 (Drummond & Rambaut, 2007). The divergence times of each lineage were estimated using the TN93+G as a site model, an uncorrelated lognormal relaxed molecular clock model, and a birth–death speciation tree prior (Ritchie et al., 2017). The mutation rate of 1% per million years ago (Mya) was adopted based on the phylogeography studies with the *Cytb* gene in cyprinid fish (Durand et al., 2002). Bayesian Markov Chain Monte Carlo (MCMC) analyses were performed for 15 million generations while sampling every 5,000^th^ tree, and the first 10% of the trees sampled were treated as burn‐in. Subsequently, the estimates and convergence of effective sample size (ESS) for all parameters larger than 200 were checked with Tracer v1.7.1 (Rambaut et al., 2018), and all resulting trees were combined with LogCombiner v1.7.3 (Drummond & Rambaut, 2007). Finally, a maximum credibility tree was produced using TreeAnnotator v1.5.3 (Drummond & Rambaut, 2007), visualized, and annotated in FigTree v1.4.2 (Rambaut, 2014).

#### 2.4.5 Demographic history

Three methods were used with the haplotype dataset to trace the demographic history. Firstly, the neutrality test between the values of Tajima’s *D* (Tajima, 1989) and Fu’s *Fs* (Fu & Li, 1993) was used to test for neutral evolution in Arlequin v3.5.1.2. Subsequently, the mismatch distribution between the values of the sum of squared deviations (SSD) and Harpending’s raggedness index (HRI) was used to test for signals of demographic expansion using Arlequin v3.5.1.2 (Harpending, 1994). Meanwhile, the beginning time of expansion (t) was calculated following a formula (t = tau/4uk, the value of tau is expansion parameter, generated by mismatch distribution with Arlequin v3.5.1.2, the value of u is the mutation rate per nucleotide, and the value of k is the length of nucleotide sequences) (Rogers & Harpending, 1992). Finally, the Bayesian skyline plot (BSP) analysis was performed with strict clock estimation using the TN93+G substitution model with a mutation rate of 1% per Mya and 15 million generations to describe demographic history by assessing the variation between time and ESS in BEAST v1.6.1 and Tracer v1.7.1.

## 3 RESULTS

### 3.1 Genetic diversity

A total of 464 sequences of *R*. *lagowskii* from 48 geographic localities were obtained (Figure 2). The sequence alignment provided a dataset matrix of a 540‐bp region, of which 115 bp (21.3%) were parsimony informative sites. Base frequency was biased with the AT content reaching 54.8%. A total of 69 haplotypes were identified and included 45 unique haplotypes and 24 shared haplotypes from distinct geographical localities samples (Table 1). The mean ratio of the number of synonymous and nonsynonymous substitutions per site (dN/dS) was 0.028 (see supplemental material Table S1), indicating purifying selection.

The haplotype diversity (*h*) and nucleotide diversity (*π*) across the samples were obtained (Table 1). The highest values of *h* and *π* were analyzed in the two localities (population code: BD and XYG) samples, respectively, while the lowest values of *h* and *π* were obtained in four localities samples (population code: GJZ, HZZ, LYG, and SZB). In addition, the most common haplotype (Hap10) was distributed in ten localities and were accounted for in 18.5% of the individuals (86 of 464), including 75 samples in the Han River, a sample in the Wei River, and ten samples in the Jialing River, respectively. Interestingly, the relationship between the genetic diversity and the population latitude and longitude was uncorrelated.

### 3.2 Phylogenetic and network analyses

**FIGURE 3:**
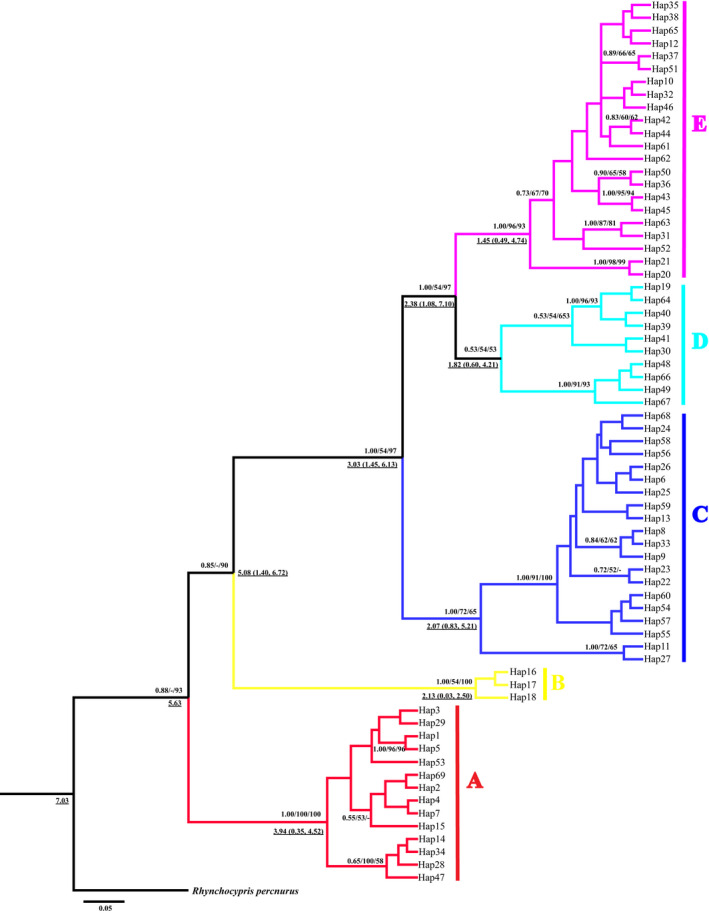
Bayesian inference tree between haplotypes based on *Cytb* sequences of *Rhynchocypris lagowskii*. The numbers above nodes are Bayesian posterior probabilities, maximum likelihood (ML), and maximum parsimony (MP) bootstrap values, respectively (above 50% are shown). The five lineages are differentiated by different colors (red, (a); yellow, (b); blue, (c); cyan, (d); purple, (e)). Estimated divergent dates in Mya are given in numbers down nodes with underline

**FIGURE 4:**
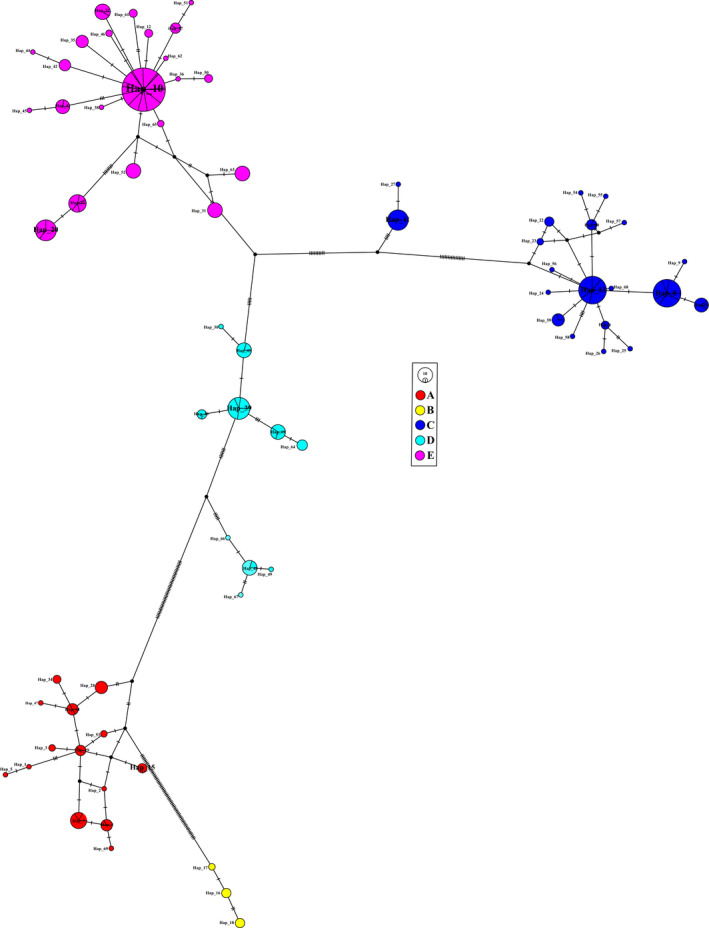
Median‐joining network for all haplotypes of *Rhynchocypris lagowskii* based on the *Cytb* gene. Each cross‐hatched line represents one base‐pair difference between haplotypes, black dots are inferred missing haplotypes, and the haplotype frequency and proportion are relative to the size and split‐line of the circle. The five different colors correspond to lineages as in Figure 3

The phylogenetic analysis found that the identified haplotypes grouped into five distinct lineages (A‐E; Figure 3). The phylogenetic trees (BI, ML, and MP) based on the haplotype sequences were congruent topologies and the different lineages did not completely correspond to localities in the Qinling Mountains (Figure 2) (the BI tree showed in Figure 3). The network analysis revealed similar results with the phylogenetic trees (Figure 4). All shared haplotypes were connected in a star‐like manner with some dominant haplotypes such as Hap10, Hap13, and Hap39. Also, all lineages in the network diagram do not completely correspond to sample localities.

### 3.3 Population genetic structure

**FIGURE 5:**
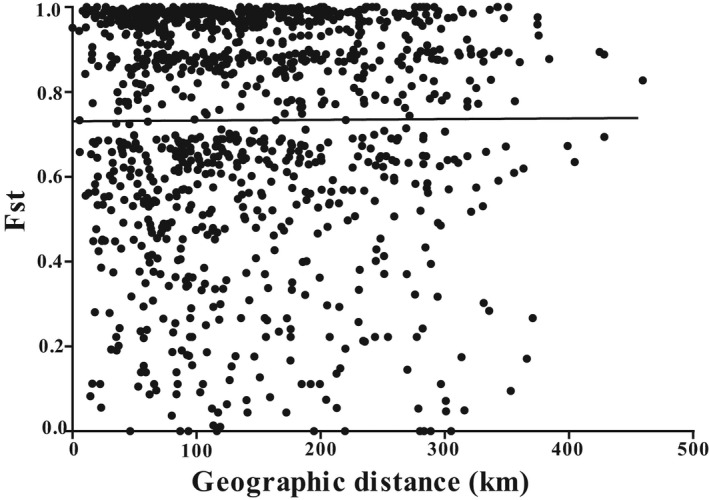
Plots of differentiation estimates of the pairwise Fst values against the geographic distance (km) between populations within the *Cytb* dataset of *Rhynchocypris lagowskii*. The linear regression overlays the scatter plots (R^2 ^= 0.00003154, *p *= .8520)

The mean genetic distance among our defined lineages ranged from 3.5% to 13.9% in the Qinling Mountains (Table 2). The lowest value was found between the lineages C and E samples, whereas the largest value occurred between the lineages C and D samples. Subsequently, the analysis of molecular variances (AMOVA) indicated that the highest proportion of genetic variation (45.5%) was attributed to the differentiation between populations, whereas the lowest proportion of genetic variation (10.5%) was attributed to the differentiation among our defined lineages (A‐E). Meanwhile, all the variance values of fixation indices were significant, showing that most of the genetic variation was a partition between populations rather than by among our defined lineages in the Qinling Mountains (Table 3). Furthermore, there was no significant correlation between the pairwise Fst values of the individuals from localities and their geographic distances (R^2 ^= 0.00003154, *p *= .852) (Figure 5), rejecting the IBD model.

**TABLE 2:** Mean genetic distance for the *Cytb* haplotypes between lineages of *Rhynchocypris lagowskii* based on the uncorrected psdistances model

Lineages	A	B	C	D	E
A					
B	0.105				
C	0.108	0.061			
D	0.126	0.131	0.139		
E	0.095	0.052	0.035	0.131	

**TABLE 3:** Results of hierarchical analysis of molecular variance (AMOVA) based on the haplotypes of *Rhynchocypris lagowskii*

Source of variation	Degree of freedom	Sum of squares	Variance components	Percentage of variation	Fixation indices
Among groups	4	26	0.0516 Va	10.5	Fct*=0.105
Among populations	43	102	0.223 Vb	45.5	Fsc*=0.508
Within populations	416	90	0.216 Vc	44.0	Fst*=0.440
Total	463	218	0.491		

Note

Significant level *p < .01.

### 3.4 Divergence time estimation

Coalescent techniques were applied to estimate the time to the most recent common ancestor (TMRCA) and divergence times of the five lineages. TMRCA of the whole ingroup dated to 7.03 Mya. The divergence times among lineages diverged from 5.63 Mya to 2.38 Mya (Figure 3). All divergence times occurred during the Late Miocene to the Early Pleistocene.

### 3.5 Demographic history

**FIGURE 6:**
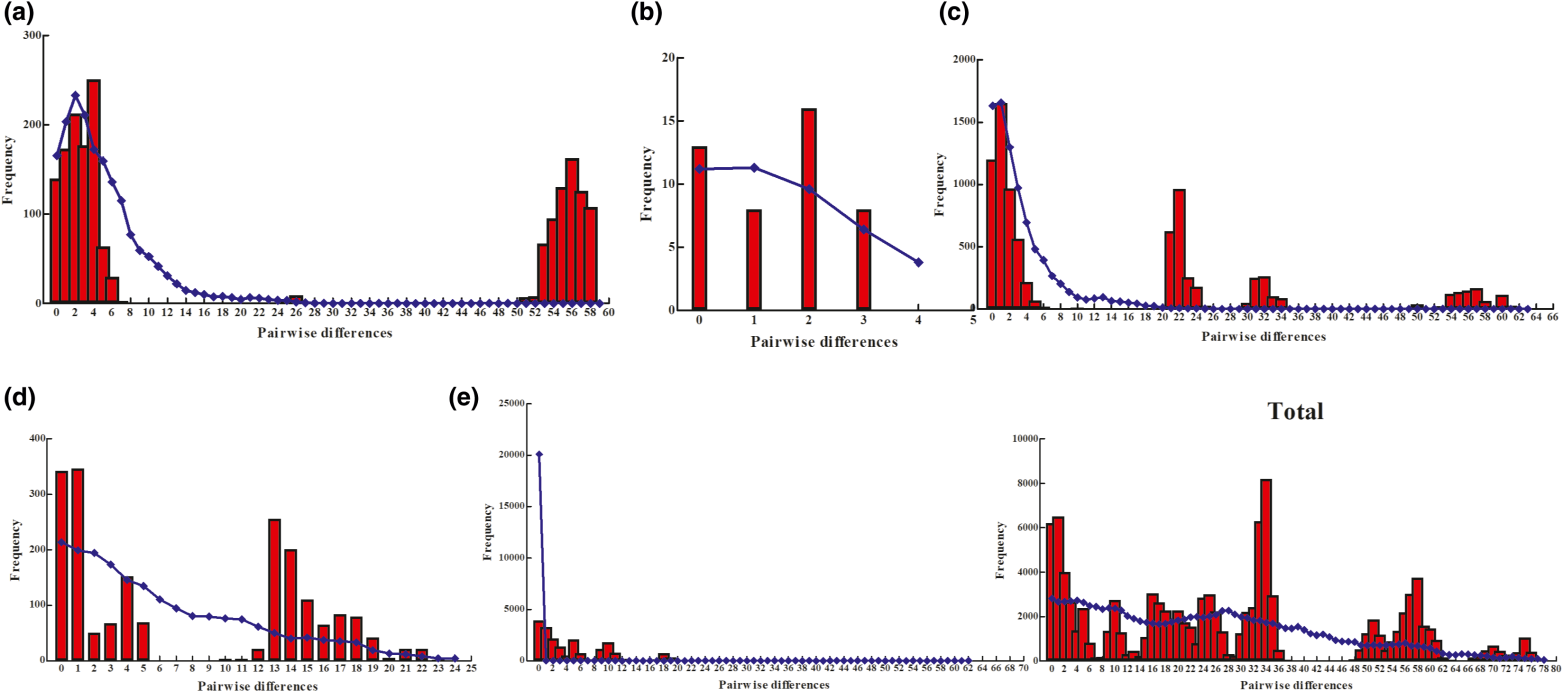
Mismatch distributions for each lineage and the total samples of *Rhynchocypris lagowskii*. The observed pairwise differences are shown as red bars and the simulated values under the sudden expansion model are blue solid lines

**FIGURE 7:**
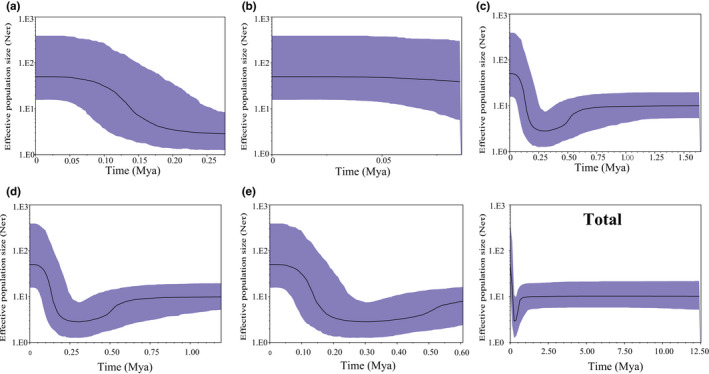
Bayesian skyline plots of historical demography for each lineage and the total samples of *Rhynchocypris lagowskii*. The solid line represents the median value of the population size and the dashed lines represent the 95% credible intervals. The X‐axis represents time using a mutation rate of 1% per million years ago (Mya), and the Y‐axis represents the effective population size

The neutrality test showed population stability except for lineage E (Table 4). However, the mismatch distribution demonstrated that the values of the SSD and HRI index of all individual lineages, except lineage E and when all lineages were analyzed collectively, did not reject the hypothesis of sudden expansion. In addition, lineage E was unimodal, showing population expansion, whereas other individual lineages and the lineages collectively were multimodal, rejecting population expansion and suggesting stability (Figure 6). Meanwhile, the beginning time of expansion (t) of all the individual lineages and the lineages collectively, except lineage E, was during the Early to the Late Pleistocene before the Last Glacial Maximum (LGM, 0.023–0.018 Mya) (Table 4). In addition, the BSP suggested that the effective population size of *R*. *lagowskii* for each lineage and the lineages collectively, except lineage B, increased rapidly approximately 0.30 Mya during the Middle to the Late Pleistocene, while lineages C to E and all the lineages collectively, underwent a slight decline from 0.70–0.30 Mya during the Middle Pleistocene (Figure 7). Therefore, lineage E underwent expansion in the demographic scenarios based on three methods.

**TABLE 4:** Statistics for the genetic diversity, neutrality test, mismatch analysis and the time of the expansion based on lineages of the *Cytb* haplotypes of *Rhynchocypris lagowskii*

Lineages	h	π	HRI	SSD	Tajima’s *D*	Fu’s *Fs*	tau	t (Mya)
A	0.921±0.017	0.177±0.0873	0.0212	0.0429	1.73	10.3	1.34	0.0621
B	0.711± 0.086	0.0105±0.00792	0.107	0.0270	1.23	1.19	2.33	0.108
C	0.857± 0.018	0.118±0.0575	0.0304	0.0415	−0.220	3.77	0.232	0.089
D	0.824±0.033	0.0477±0.0246	0.0468	0.0463	0.692	3.95	16.1	0.743
E	0.805±0.026	0.0402±0.0215	0.0272	0.719**	−1.77**	−1.00	0.000	0.000
Total	0.942±0.006	0.0514±0.00187	0.00855*	0.0149	1.12	2.900	25.8	1.19

Note

Significant level *p < .05, **p < .01.

Abbreviations: h, haplotype diversity; HRI, Harpending’s raggedness index; Mya, million years ago; SSD, sum of squared deviation; t, beginning time of expansion; tau, expansion parameter; π, nucleotide diversity.

## 4 DISCUSSION

### 4.1 The geological barrier drove the differentiation and phylogeographic pattern among species in the Qinling Mountains

The Qinling Mountains represent a natural boundary between northern and southern China. The rapid uplift of these mountains was mainly influenced by the Qinghai–Tibet Plateau movement which began from the Miocene to the Holocene, especially starting at the Early Pleistocene (Zhang & Fang, 2012), and the changing topography likely resulted in population differentiation for a number of terrestrial and freshwater fauna (Li, 1981; Zhang, 2011). Previous studies have shown that these mountains have played an important role as a geographical barrier in shaping the significant phylogeographic patterns of many species with a low dispersal ability, including amphibian, fish, and mammal species and may have led to the fragmentation of populations by vicariance (Hardouin et al., 2018; Huang et al., 2017; Li et al., 2021; Liu et al., 2015; Meng et al., 2014; Shao et al., 2019; Wang et al., 2012, 2013; Yu et al., 2014).

Subsequently, our study found that the phylogenetic trees, network, and AMOVA yielded well‐differentiated lineages. A clear phylogeographic pattern was observed with some shared haplotypes of *R*. *lagowskii* across geographic locations. The current pattern was likely shaped by lower dispersal ability, wide sampling, and past vicariance (genetic isolation among adjacent areas by natural barriers) followed by a dispersal and secondary contact. The mean genetic distance among haplotypes of *R*. *lagowskii* ranged from 3.5% to 13.9% (overall mean distance, 6.5%). This was higher than that found in amphibians *S*. *ningshanensis* (2.4% to 4.2%) and *B*. *tibetanus* (0.0% to 3.7%) but similar to values found in other fish and amphibian species, including *R*. *oxycephalus* (6.5% to 7.4%) and *Odorrana schmackeri* (3.4% to 21.1%) in the Qinling Mountains (Huang et al., 2017; Li et al., 2015; Meng et al., 2014; Yu et al., 2014). The AMOVA indicated that geographic structuring and the percentage of variation were the lowest among lineages and the highest between populations. Thus, this pattern corresponds to the high genetic differentiation among populations of *R*. *lagowskii*, which is similar to results obtained in another amphibian and fish species in this region owing to the lower dispersal ability with the restricted gene flow and high differentiation (Blasco‐Costa et al., 2012; Hardouin et al., 2018; Meng et al., 2014; Shao et al., 2019; Yu et al., 2014).

Furthermore, there was no significant positive correlation between the pairwise Fst values and geographic distance (km) of *R*. *lagowskii* and this lacked IBD. This is similar to results from some other amphibian and mussel species (Li et al., 2015; Liu et al., 2017; Wang et al., 2013). Our study showed the current phylogeographical pattern of *R*. *lagowskii* was likely affected by past vicariance (genetic isolation among adjacent areas by natural barriers) followed by dispersal and secondary contact.

Therefore, based on phylogenetic trees, network, mean genetic distance, AMOVA, and IBD analyses of *R*. *lagowskii*, we found that as *R*. *lagowskii* is primarily found in clear cold freshwater from the midstream to upstream in East Asia (Kang et al., 2000; Nishida et al., 2015; Zhang & Chen, 1997), the specific ecological upstream distribution results in much smaller population size and higher differentiation between populations (Yu et al., 2014). In addition, the rapid uplift of the Qinling Mountains during the Miocene to the Holocene, especially starting at the Early Pleistocene modified the topography of this area, potentially creating more isolated geographic pockets of suitable habitat (Zhang & Fang, 2012), and river capture related to the new tectonic movements also occurred and accelerated fish dispersal based on some well‐supported phylogenetic lineages and the values of genetic diversity between the samples from watershed localities in this region (Zhang, 1962). So it is stated that vicariance and uplift promote differentiation but river capture promotes dispersal (Albert & Crampton, 2010; Albert et al., 2017; Zhang & Chen, 1997; Zhang & Fang, 2012). Also, upstream barriers might limit gene flow (Blasco‐Costa et al., 2012).

### 4.2 Genetic diversity change during the glaciation and purifying selection

The *R*. *lagowskii* showed high levels of genetic diversity with many unique haplotypes and with some haplotypes being found across wide geographic regions indicating limited gene flow.

However, there were three unique haplotypes and four shared haplotypes in ten of the sampled localities and these populations had extremely low levels of genetic diversity. This is different from the results that found eleven unique haplotypes and one shared haplotype in twelve localities populations of *R*. *oxycephalus* (Yu et al., 2014). The genetic diversity loss across these low diversity localities is likely the result of genetic drift, bottleneck, founder effect, habitat fragmentation, inbreeding, and gene flow deficiency (Hunter & Gibbs, 2006). Our results are in slight contrast to what has been observed for haplotypes of the congeneric *R*. *oxycelphalus*. This species shows a more widespread geographic distribution with successive sampling, where the haplotypes per locality were mostly unique and not widely distributed with limited sampling in the Qinling Mountains (Yu et al., 2014).

Some of the genetic patterns observed in *R*. *lagowskii* may be the results of various refugia that were available during the LGM. During this period, ice sheet or glaciation extended south to the high latitude and altitude regions in Europe and North America at the LGM (Hewitt, 1996). The fish and vertebrate species expanded mainly from southern refugia in more recent interglacials, reducing genetic diversity, and forming distinct phylogeographical patterns after the LGM (Hewitt, 1996; Rowe et al., 2004). The high altitude region in the Qinling Mountains was also affected by Taibai glaciation (0.019 Ma) (Shi, 2002); however, the genetic diversity of *R*. *lagowskii* did not show the significant latitude and altitude decreasing trend often observed with other mussel, fish, amphibian, and mammal species in the Qinling Mountains and other regions of China (Hardouin et al., 2018; Li et al., 2021; Liu et al., 2015, 2017; Shao et al., 2019; Wang et al., 2012; Xue et al., 2017; Yu et al., 2014). In addition, populations with high haplotype diversity and low nucleotide diversity, such as what has previously been found in *B*. *lenok tsinlingensis*, and *B*. *tsinlingensis*, and populations with a star‐shaped haplotype network, such as that observed for *R*. *lagowskii*, are indicative of a classic postglacial expansion after a period of low effective population size, with rapid population growth enhancing the retention of new mutations, accumulating haplotype diversity, but lacking enough time to accumulate nucleotide diversity (Grant & Bowen, 1998; Hewitt, 1996; Liu et al., 2015; Shao et al., 2019).

We tested whether the population of *R*. *lagowskii* underwent natural selection through the mean dN/dS value of the mitochondrial coding protein gene (*Cytb*). The dN/dS value from coding protein genes has been used to detect patterns of selection in molecular evolution (Kimura, 1977; Yang & Bielawski, 2000). Our study also showed the mean dN/dS value was 0.028 for *Cytb*. Past work evaluating neural crest‐associated genes and mitochondrial protein‐coding genes in fish have suggested that when the dN/dS value is below 0.1 this suggest the genes are under strong purifying selection and slow evolution, safeguarding the biological function of proteins against deleterious mutations (Kratochwil et al., 2015; Lu et al., 2019; Rand & Kann, 1998). Therefore, the populations of *R*. *lagowskii* probably underwent purifying selection with slow evolution and rapid adaptation with an incomplete mitochondrial gene and a relatively low value of nucleotide diversity (*π*) against nonsynonymous substitutions often observed with 13 mitochondrial oxidative phosphorylation (OXPHOS) genes among other cyprinid species (Lu et al., 2019). More genetic data would be needed to test this conclusion in further study.

### 4.3 Population divergence and demographic history

The divergence time among all lineages of *R*. *lagowskii* was during the Late Miocene to the Early Pleistocene and is similar to results from some amphibian and fish species in this region (Hardouin et al., 2018; Huang et al., 2017; Li et al., 2015; Yu et al., 2014), representing an ancient separation. Previous studies showed the phylogeographic patterns of some amphibian and fish species were profoundly influenced by climate oscillations and tectonic barriers with the rapid uplift of the Qinling Mountains during this period (Gao et al., 2012; Meng et al., 2014; Wang et al., 2013; Yu et al., 2014).

The neutrality test, except lineage E, showed population stability in this region with other amphibian, fish, and mammal species (Hu et al., 2021; Meng et al., 2014; Yu et al., 2014). However, the mismatch distribution, except lineage E, did not reject the hypothesis of sudden expansion. In addition, lineage E was unimodal and showed expansion, whereas others were stable. The beginning time of expansion (t), except lineage E, was during the Early to the Late Pleistocene before the LGM following the rapid uplift of these mountains (Zhang & Fang, 2012). Subsequently, the BSP suggested rapid expansion, except lineage B, during the Middle to the Late Pleistocene along with warming temperatures and corresponded to end of Lushan glaciation (0.2 – 0.4 Ma) and this similar to what has been found in other amphibians and fish in this region (He et al., 1992; Huang, 1982; Wang et al., 2013; Yu et al., 2014). In addition, lineages C to E and all lineages collectively underwent a slight decline during the Middle Pleistocene, and other amphibian and fish species underwent a sharp contraction during the Late Pleistocene after the LGM in this region and their distributions were affected by the climatic oscillation (Meng et al., 2014; Yu et al., 2014).

Therefore, the results of demographic history based on the neutrality test, mismatch distribution, and BSP analyses showed that all lineages collectively were likely stable during the Pleistocene, and lineage E probably underwent a slight contraction during the Middle Pleistocene and rapid expansion from the Middle to the Late Pleistocene. Recently, the specimens of *R*. *lagowskii* from lineage E are mainly distributed upstream of the Han River and Wei River from the Middle to Western Qinling Mountains and adapt the cold climate with congeneric species *R*. *oxycephalus* (Yu et al., 2014).

## 5 CONCLUSIONS

Our studies suggest the current phylogeographical pattern of *R*. *lagowskii* was likely shaped by the tectonic changes and climatic oscillation during the Late Miocene to the Early Pleistocene in the Qinling Mountains. The total samples were stable during the Pleistocene, while lineage E probably underwent slight contraction during the Middle Pleistocene and rapid expansion from the Middle to the Late Pleistocene. A further study employing extensive sampling with larger geographical intervals, more specimens, complete mtDNA sequences of *Cytb* gene, and multiple molecular markers might provide more insight into the phylogeographical pattern of this species across the whole geographical range. Finally, the phylogeographical pattern of *R*. *lagowskii* helps maintain its genetic diversity and further conservation in China.

## AUTHOR CONTRIBUTION

**Tao Chen:** Data curation (lead); Formal analysis (lead); Investigation (equal); Methodology (equal); Software (lead); Writing – original draft (lead); Writing – review & editing (lead). **Li Jiao:** Investigation (equal); Methodology (equal). **Lili Ni:** Investigation (equal); Methodology (equal).

## CONFLICT OF INTEREST

The authors declare that they have no competing interests.

## Supporting information

Table S1

## Data Availability

The haplotype DNA sequences were deposited in GenBank under accession numbers MW831313‐MW831381. The data have been uploaded into Dryad under the following https://doi.org/10.5061/dryad.70rxwdc0v.
