# Supplementary material for: The phylogeographical pattern of the Amur minnow Rhynchocypris lagowskii (Cypriniformes: Cyprinidae) in the Qinling Mountains
Source: Ecol Evol. 2022 May 15;12(5):e8924. doi: 10.1002/ece3.8924 (PMC9108317; doi:10.1002/ece3.8924)
Supplement: Supplementary file 1 — Table S1 [file ECE3-12-e8924-s001.docx]

**Supplemental material Table S1**

| TABLE S1. Results of maximum likelihood analysis of the natural selection codon-by-codon via HyPhy based on the Cytb gene of Rhynchocypris lagowskii | | | | | | | | | | | |
| --- | --- | --- | --- | --- | --- | --- | --- | --- | --- | --- | --- |
| Codon | Codon Start | Triplet | Syn (s) | Nonsyn (n) | Syn sites (S) | Nonsyn sites (N) | dS | dN | dN-dS | P-value | Normalized dN-dS |
| 1 | 1 | ATT | 0 | 1 | 0.881 | 2.119 | 0.000 | 0.472 | 0.472 | 0.706 | 1.012 |
| 2 | 4 | ACC | 1 | 0 | 1.000 | 2.000 | 1.000 | 0.000 | -1.000 | 1.000 | -2.145 |
| 3 | 7 | CAA | 4 | 0 | 0.874 | 1.337 | 4.579 | 0.000 | -4.579 | 1.000 | -9.819 |
| 4 | 10 | ATC | 2 | 0 | 0.803 | 2.197 | 2.490 | 0.000 | -2.490 | 1.000 | -5.340 |
| 5 | 13 | CTC | 0 | 0 | 1.000 | 2.000 | 0.000 | 0.000 | 0.000 | N/A | 0.000 |
| 6 | 16 | ACG | 1 | 0 | 1.000 | 1.918 | 1.000 | 0.000 | -1.000 | 1.000 | -2.145 |
| 7 | 19 | GGA | 3 | 0 | 1.000 | 1.102 | 3.000 | 0.000 | -3.000 | 1.000 | -6.434 |
| 8 | 22 | TTA | 2 | 0 | 1.757 | 1.202 | 1.138 | 0.000 | -1.138 | 1.000 | -2.441 |
| 9 | 25 | TTC | 2 | 0 | 0.864 | 2.136 | 2.314 | 0.000 | -2.314 | 1.000 | -4.963 |
| 10 | 28 | CTA | 2 | 0 | 1.790 | 1.210 | 1.118 | 0.000 | -1.118 | 1.000 | -2.397 |
| 11 | 31 | GCT | 2 | 0 | 1.000 | 2.000 | 2.000 | 0.000 | -2.000 | 1.000 | -4.289 |
| 12 | 34 | ATA | 2 | 0 | 0.875 | 2.084 | 2.286 | 0.000 | -2.286 | 1.000 | -4.902 |
| 13 | 37 | CAT | 1 | 0 | 0.801 | 2.199 | 1.248 | 0.000 | -1.248 | 1.000 | -2.677 |
| 14 | 40 | TAC | 2 | 0 | 0.800 | 2.000 | 2.499 | 0.000 | -2.499 | 1.000 | -5.360 |
| 15 | 43 | ACC | 0 | 0 | 1.000 | 2.000 | 0.000 | 0.000 | 0.000 | N/A | 0.000 |
| 16 | 46 | TCT | 0 | 0 | 1.000 | 2.000 | 0.000 | 0.000 | 0.000 | N/A | 0.000 |
| 17 | 49 | GAC | 1 | 0 | 0.800 | 2.200 | 1.250 | 0.000 | -1.250 | 1.000 | -2.681 |
| 18 | 52 | ATT | 0 | 0 | 0.880 | 2.120 | 0.000 | 0.000 | 0.000 | N/A | 0.000 |
| 19 | 55 | TCC | 1 | 0 | 1.000 | 1.985 | 1.000 | 0.000 | -1.000 | 1.000 | -2.145 |
| 20 | 58 | ACT | 1 | 0 | 1.000 | 1.991 | 1.000 | 0.000 | -1.000 | 1.000 | -2.145 |
| 21 | 61 | GCA | 3 | 0 | 1.000 | 2.000 | 3.000 | 0.000 | -3.000 | 1.000 | -6.434 |
| 22 | 64 | TTT | 2 | 0 | 0.831 | 2.169 | 2.406 | 0.000 | -2.406 | 1.000 | -5.161 |
| 23 | 67 | TCA | 0 | 0 | 1.000 | 1.871 | 0.000 | 0.000 | 0.000 | N/A | 0.000 |
| 24 | 70 | TCA | 0 | 0 | 1.000 | 1.871 | 0.000 | 0.000 | 0.000 | N/A | 0.000 |
| 25 | 73 | GTC | 4 | 0 | 1.000 | 2.000 | 4.000 | 0.000 | -4.000 | 1.000 | -8.578 |
| 26 | 76 | GCC | 1 | 0 | 1.000 | 2.000 | 1.000 | 0.000 | -1.000 | 1.000 | -2.145 |
| 27 | 79 | CAC | 0 | 0 | 0.790 | 2.210 | 0.000 | 0.000 | 0.000 | N/A | 0.000 |
| 28 | 82 | ATC | 1 | 0 | 0.800 | 2.200 | 1.250 | 0.000 | -1.250 | 1.000 | -2.681 |
| 29 | 85 | TGC | 0 | 0 | 0.790 | 2.210 | 0.000 | 0.000 | 0.000 | N/A | 0.000 |
| 30 | 88 | CGA | 1 | 0 | 1.000 | 1.984 | 1.000 | 0.000 | -1.000 | 1.000 | -2.145 |
| 31 | 91 | GAC | 1 | 0 | 0.839 | 2.161 | 1.191 | 0.000 | -1.191 | 1.000 | -2.555 |
| 32 | 94 | GTT | 1 | 0 | 1.000 | 2.000 | 1.000 | 0.000 | -1.000 | 1.000 | -2.145 |
| 33 | 97 | AAC | 1 | 0 | 0.869 | 2.131 | 1.151 | 0.000 | -1.151 | 1.000 | -2.468 |
| 34 | 100 | TAT | 2 | 0 | 0.831 | 2.000 | 2.407 | 0.000 | -2.407 | 1.000 | -5.163 |
| 35 | 103 | GGC | 0 | 0 | 1.000 | 2.000 | 0.000 | 0.000 | 0.000 | N/A | 0.000 |
| 36 | 106 | TGA | 0 | 0 | 0.848 | 1.176 | 0.000 | 0.000 | 0.000 | N/A | 0.000 |
| 37 | 109 | CTA | 1 | 0 | 1.699 | 1.301 | 0.589 | 0.000 | -0.589 | 1.000 | -1.262 |
| 38 | 112 | ATC | 0 | 0 | 0.790 | 2.210 | 0.000 | 0.000 | 0.000 | N/A | 0.000 |
| 39 | 115 | CGA | 1 | 0 | 1.000 | 1.871 | 1.000 | 0.000 | -1.000 | 1.000 | -2.145 |
| 40 | 118 | AGC | 0 | 0 | 0.790 | 2.000 | 0.000 | 0.000 | 0.000 | N/A | 0.000 |
| 41 | 121 | CTG | 5 | 0 | 1.790 | 1.210 | 2.794 | 0.000 | -2.794 | 1.000 | -5.992 |
| 42 | 124 | CAC | 1 | 0 | 0.869 | 2.131 | 1.151 | 0.000 | -1.151 | 1.000 | -2.468 |
| 43 | 127 | GCC | 1 | 1 | 1.000 | 2.000 | 1.000 | 0.500 | -0.500 | 0.889 | -1.072 |
| 44 | 130 | AAC | 1 | 0 | 0.816 | 2.184 | 1.226 | 0.000 | -1.226 | 1.000 | -2.629 |
| 45 | 133 | GGA | 2 | 0 | 1.000 | 1.102 | 2.000 | 0.000 | -2.000 | 1.000 | -4.289 |
| 46 | 136 | GCA | 0 | 0 | 1.000 | 2.000 | 0.000 | 0.000 | 0.000 | N/A | 0.000 |
| 47 | 139 | TCC | 1 | 0 | 1.000 | 2.000 | 1.000 | 0.000 | -1.000 | 1.000 | -2.145 |
| 48 | 142 | TTC | 0 | 0 | 0.790 | 2.210 | 0.000 | 0.000 | 0.000 | N/A | 0.000 |
| 49 | 145 | TTT | 1 | 0 | 0.801 | 2.199 | 1.248 | 0.000 | -1.248 | 1.000 | -2.677 |
| 50 | 148 | TTC | 1 | 0 | 0.790 | 2.210 | 1.266 | 0.000 | -1.266 | 1.000 | -2.715 |
| 51 | 151 | ATC | 0 | 0 | 0.790 | 2.210 | 0.000 | 0.000 | 0.000 | N/A | 0.000 |
| 52 | 154 | TGC | 1 | 0 | 0.869 | 2.131 | 1.151 | 0.000 | -1.151 | 1.000 | -2.468 |
| 53 | 157 | ATT | 0 | 0 | 0.880 | 2.120 | 0.000 | 0.000 | 0.000 | N/A | 0.000 |
| 54 | 160 | TAT | 1 | 0 | 0.866 | 2.000 | 1.154 | 0.000 | -1.154 | 1.000 | -2.476 |
| 55 | 163 | ATA | 2 | 0 | 0.854 | 2.106 | 2.343 | 0.000 | -2.343 | 1.000 | -5.025 |
| 56 | 166 | CAC | 0 | 0 | 0.790 | 2.210 | 0.000 | 0.000 | 0.000 | N/A | 0.000 |
| 57 | 169 | ATT | 0 | 0 | 0.880 | 2.120 | 0.000 | 0.000 | 0.000 | N/A | 0.000 |
| 58 | 172 | GCA | 3 | 0 | 1.000 | 2.000 | 3.000 | 0.000 | -3.000 | 1.000 | -6.434 |
| 59 | 175 | CGG | 3 | 0 | 1.000 | 1.871 | 3.000 | 0.000 | -3.000 | 1.000 | -6.434 |
| 60 | 178 | GGC | 1 | 1 | 1.000 | 2.000 | 1.000 | 0.500 | -0.500 | 0.889 | -1.073 |
| 61 | 181 | CTA | 3 | 0 | 1.789 | 1.210 | 1.676 | 0.000 | -1.676 | 1.000 | -3.595 |
| 62 | 184 | TAT | 0 | 0 | 0.880 | 2.000 | 0.000 | 0.000 | 0.000 | N/A | 0.000 |
| 63 | 187 | TAT | 3 | 0 | 0.869 | 2.000 | 3.453 | 0.000 | -3.453 | 1.000 | -7.404 |
| 64 | 190 | GGC | 1 | 0 | 1.000 | 1.897 | 1.000 | 0.000 | -1.000 | 1.000 | -2.145 |
| 65 | 193 | TCG | 3 | 0 | 1.000 | 1.871 | 3.000 | 0.000 | -3.000 | 1.000 | -6.434 |
| 66 | 196 | TAT | 1 | 0 | 0.801 | 2.000 | 1.248 | 0.000 | -1.248 | 1.000 | -2.677 |
| 67 | 199 | CTT | 1 | 0 | 1.000 | 2.000 | 1.000 | 0.000 | -1.000 | 1.000 | -2.145 |
| 68 | 202 | TAT | 0 | 0 | 0.880 | 2.000 | 0.000 | 0.000 | 0.000 | N/A | 0.000 |
| 69 | 205 | AAA | 2 | 0 | 0.853 | 1.242 | 2.345 | 0.000 | -2.345 | 1.000 | -5.028 |
| 70 | 208 | GAG | 1 | 1 | 0.898 | 2.069 | 1.113 | 0.483 | -0.630 | 0.908 | -1.351 |
| 71 | 211 | ACC | 1 | 0 | 1.000 | 2.000 | 1.000 | 0.000 | -1.000 | 1.000 | -2.145 |
| 72 | 214 | TGA | 0 | 0 | 0.848 | 1.176 | 0.000 | 0.000 | 0.000 | N/A | 0.000 |
| 73 | 217 | AAT | 1 | 0 | 0.870 | 2.130 | 1.149 | 0.000 | -1.149 | 1.000 | -2.465 |
| 74 | 220 | ATT | 2 | 0 | 0.857 | 2.143 | 2.335 | 0.000 | -2.335 | 1.000 | -5.008 |
| 75 | 223 | GGT | 0 | 0 | 1.000 | 2.000 | 0.000 | 0.000 | 0.000 | N/A | 0.000 |
| 76 | 226 | GTT | 1 | 0 | 1.000 | 2.000 | 1.000 | 0.000 | -1.000 | 1.000 | -2.145 |
| 77 | 229 | GTA | 2 | 3 | 0.907 | 2.093 | 2.206 | 1.433 | -0.773 | 0.834 | -1.658 |
| 78 | 232 | CTA | 2 | 0 | 1.790 | 1.210 | 1.118 | 0.000 | -1.118 | 1.000 | -2.397 |
| 79 | 235 | CTT | 1 | 0 | 1.000 | 2.000 | 1.000 | 0.000 | -1.000 | 1.000 | -2.145 |
| 80 | 238 | CTC | 3 | 0 | 1.206 | 1.794 | 2.488 | 0.000 | -2.488 | 1.000 | -5.335 |
| 81 | 241 | TTA | 2 | 0 | 1.744 | 1.177 | 1.146 | 0.000 | -1.146 | 1.000 | -2.459 |
| 82 | 244 | GTT | 6 | 2 | 1.000 | 1.998 | 6.002 | 1.001 | -5.001 | 0.997 | -10.725 |
| 83 | 247 | ATG | 5 | 1 | 0.862 | 2.098 | 5.803 | 0.477 | -5.326 | 0.999 | -11.422 |
| 84 | 250 | GCA | 1 | 0 | 1.000 | 2.000 | 1.000 | 0.000 | -1.000 | 1.000 | -2.145 |
| 85 | 253 | ACG | 2 | 0 | 1.000 | 1.918 | 2.000 | 0.000 | -2.000 | 1.000 | -4.289 |
| 86 | 256 | GCC | 0 | 0 | 1.000 | 2.000 | 0.000 | 0.000 | 0.000 | N/A | 0.000 |
| 87 | 259 | TTT | 0 | 0 | 0.880 | 2.120 | 0.000 | 0.000 | 0.000 | N/A | 0.000 |
| 88 | 262 | GTG | 5 | 1 | 1.002 | 1.998 | 4.992 | 0.500 | -4.492 | 0.999 | -9.633 |
| 89 | 265 | GGT | 1 | 0 | 1.000 | 2.000 | 1.000 | 0.000 | -1.000 | 1.000 | -2.145 |
| 90 | 268 | TAT | 1 | 0 | 0.870 | 2.000 | 1.149 | 0.000 | -1.149 | 1.000 | -2.465 |
| 91 | 271 | GTT | 2 | 0 | 1.000 | 2.000 | 2.000 | 0.000 | -2.000 | 1.000 | -4.289 |
| 92 | 274 | CTT | 1 | 0 | 1.000 | 2.000 | 1.000 | 0.000 | -1.000 | 1.000 | -2.145 |
| 93 | 277 | CCC | 1 | 0 | 1.000 | 2.000 | 1.000 | 0.000 | -1.000 | 1.000 | -2.145 |
| 94 | 280 | TGA | 0 | 0 | 0.848 | 1.176 | 0.000 | 0.000 | 0.000 | N/A | 0.000 |
| 95 | 283 | GGA | 1 | 0 | 1.000 | 1.102 | 1.000 | 0.000 | -1.000 | 1.000 | -2.145 |
| 96 | 286 | CAG | 1 | 0 | 0.854 | 1.357 | 1.171 | 0.000 | -1.171 | 1.000 | -2.512 |
| 97 | 289 | ATA | 1 | 0 | 0.875 | 2.084 | 1.143 | 0.000 | -1.143 | 1.000 | -2.450 |
| 98 | 292 | TCT | 0 | 0 | 1.000 | 2.000 | 0.000 | 0.000 | 0.000 | N/A | 0.000 |
| 99 | 295 | TTC | 1 | 0 | 0.800 | 2.200 | 1.250 | 0.000 | -1.250 | 1.000 | -2.681 |
| 100 | 298 | TGA | 1 | 0 | 0.848 | 1.176 | 1.180 | 0.000 | -1.180 | 1.000 | -2.530 |
| 101 | 301 | GGT | 0 | 0 | 1.000 | 2.000 | 0.000 | 0.000 | 0.000 | N/A | 0.000 |
| 102 | 304 | GCC | 2 | 0 | 1.000 | 2.000 | 2.000 | 0.000 | -2.000 | 1.000 | -4.289 |
| 103 | 307 | ACG | 1 | 0 | 1.000 | 1.918 | 1.000 | 0.000 | -1.000 | 1.000 | -2.145 |
| 104 | 310 | GTC | 2 | 0 | 1.000 | 2.000 | 2.000 | 0.000 | -2.000 | 1.000 | -4.289 |
| 105 | 313 | ATT | 0 | 0 | 0.880 | 2.120 | 0.000 | 0.000 | 0.000 | N/A | 0.000 |
| 106 | 316 | ACA | 1 | 0 | 1.000 | 1.919 | 1.000 | 0.000 | -1.000 | 1.000 | -2.145 |
| 107 | 319 | AAC | 0 | 0 | 0.790 | 2.210 | 0.000 | 0.000 | 0.000 | N/A | 0.000 |
| 108 | 322 | CTA | 3 | 0 | 1.666 | 1.334 | 1.801 | 0.000 | -1.801 | 1.000 | -3.862 |
| 109 | 325 | ATA | 0 | 0 | 0.848 | 2.112 | 0.000 | 0.000 | 0.000 | N/A | 0.000 |
| 110 | 328 | TCA | 0 | 0 | 1.000 | 1.871 | 0.000 | 0.000 | 0.000 | N/A | 0.000 |
| 111 | 331 | GCA | 0 | 0 | 1.000 | 2.000 | 0.000 | 0.000 | 0.000 | N/A | 0.000 |
| 112 | 334 | GTC | 1 | 0 | 1.000 | 2.000 | 1.000 | 0.000 | -1.000 | 1.000 | -2.145 |
| 113 | 337 | CCT | 0 | 0 | 1.000 | 2.000 | 0.000 | 0.000 | 0.000 | N/A | 0.000 |
| 114 | 340 | TAC | 1 | 0 | 0.839 | 2.000 | 1.191 | 0.000 | -1.191 | 1.000 | -2.555 |
| 115 | 343 | ATA | 5 | 0 | 0.869 | 2.090 | 5.754 | 0.000 | -5.754 | 1.000 | -12.340 |
| 116 | 346 | GGT | 4 | 0 | 1.000 | 1.897 | 4.000 | 0.000 | -4.000 | 1.000 | -8.578 |
| 117 | 349 | GAC | 1 | 0 | 0.800 | 2.200 | 1.250 | 0.000 | -1.250 | 1.000 | -2.681 |
| 118 | 352 | ACC | 0 | 0 | 1.000 | 2.000 | 0.000 | 0.000 | 0.000 | N/A | 0.000 |
| 119 | 355 | CTT | 1 | 0 | 1.000 | 2.000 | 1.000 | 0.000 | -1.000 | 1.000 | -2.145 |
| 120 | 358 | GTT | 1 | 0 | 1.000 | 2.000 | 1.000 | 0.000 | -1.000 | 1.000 | -2.145 |
| 121 | 361 | CAA | 1 | 0 | 0.848 | 1.363 | 1.180 | 0.000 | -1.180 | 1.000 | -2.530 |
| 122 | 364 | TGG | 1 | 0 | 0.854 | 1.170 | 1.171 | 0.000 | -1.171 | 1.000 | -2.512 |
| 123 | 367 | ATC | 1 | 0 | 0.790 | 2.210 | 1.266 | 0.000 | -1.266 | 1.000 | -2.715 |
| 124 | 370 | TGA | 4 | 0 | 0.872 | 1.151 | 4.586 | 0.000 | -4.586 | 1.000 | -9.835 |
| 125 | 373 | GGC | 0 | 0 | 1.000 | 2.000 | 0.000 | 0.000 | 0.000 | N/A | 0.000 |
| 126 | 376 | GGC | 0 | 0 | 1.000 | 2.000 | 0.000 | 0.000 | 0.000 | N/A | 0.000 |
| 127 | 379 | TTC | 0 | 0 | 0.790 | 2.210 | 0.000 | 0.000 | 0.000 | N/A | 0.000 |
| 128 | 382 | TCC | 2 | 0 | 1.000 | 1.888 | 2.000 | 0.000 | -2.000 | 1.000 | -4.289 |
| 129 | 385 | GTA | 0 | 0 | 1.000 | 2.000 | 0.000 | 0.000 | 0.000 | N/A | 0.000 |
| 130 | 388 | GAT | 3 | 0 | 0.860 | 2.140 | 3.489 | 0.000 | -3.489 | 1.000 | -7.483 |
| 131 | 391 | AAC | 0 | 0 | 0.790 | 2.210 | 0.000 | 0.000 | 0.000 | N/A | 0.000 |
| 132 | 394 | GCA | 0 | 0 | 1.000 | 2.000 | 0.000 | 0.000 | 0.000 | N/A | 0.000 |
| 133 | 397 | ACT | 0 | 0 | 1.000 | 2.000 | 0.000 | 0.000 | 0.000 | N/A | 0.000 |
| 134 | 400 | CTC | 1 | 0 | 1.000 | 2.000 | 1.000 | 0.000 | -1.000 | 1.000 | -2.145 |
| 135 | 403 | ACA | 2 | 0 | 1.000 | 1.918 | 2.000 | 0.000 | -2.000 | 1.000 | -4.289 |
| 136 | 406 | CGA | 0 | 0 | 1.000 | 1.871 | 0.000 | 0.000 | 0.000 | N/A | 0.000 |
| 137 | 409 | TTC | 0 | 0 | 0.790 | 2.210 | 0.000 | 0.000 | 0.000 | N/A | 0.000 |
| 138 | 412 | TTT | 1 | 0 | 0.801 | 2.199 | 1.248 | 0.000 | -1.248 | 1.000 | -2.677 |
| 139 | 415 | GCC | 0 | 0 | 1.000 | 2.000 | 0.000 | 0.000 | 0.000 | N/A | 0.000 |
| 140 | 418 | TTC | 0 | 0 | 0.790 | 2.210 | 0.000 | 0.000 | 0.000 | N/A | 0.000 |
| 141 | 421 | CAC | 0 | 0 | 0.790 | 2.210 | 0.000 | 0.000 | 0.000 | N/A | 0.000 |
| 142 | 424 | TTC | 1 | 0 | 0.800 | 2.200 | 1.250 | 0.000 | -1.250 | 1.000 | -2.681 |
| 143 | 427 | CTC | 1 | 0 | 1.000 | 2.000 | 1.000 | 0.000 | -1.000 | 1.000 | -2.145 |
| 144 | 430 | CTA | 5 | 0 | 1.688 | 1.312 | 2.963 | 0.000 | -2.963 | 1.000 | -6.353 |
| 145 | 433 | CCT | 3 | 0 | 1.000 | 2.000 | 3.000 | 0.000 | -3.000 | 1.000 | -6.434 |
| 146 | 436 | TTT | 1 | 0 | 0.880 | 2.120 | 1.137 | 0.000 | -1.137 | 1.000 | -2.438 |
| 147 | 439 | ATC | 0 | 0 | 0.790 | 2.210 | 0.000 | 0.000 | 0.000 | N/A | 0.000 |
| 148 | 442 | ATT | 2 | 0 | 0.804 | 2.196 | 2.487 | 0.000 | -2.487 | 1.000 | -5.333 |
| 149 | 445 | GCC | 0 | 0 | 1.000 | 2.000 | 0.000 | 0.000 | 0.000 | N/A | 0.000 |
| 150 | 448 | GGC | 1 | 0 | 1.000 | 1.998 | 1.000 | 0.000 | -1.000 | 1.000 | -2.145 |
| 151 | 451 | GCA | 1 | 0 | 1.000 | 2.000 | 1.000 | 0.000 | -1.000 | 1.000 | -2.145 |
| 152 | 454 | ACC | 2 | 0 | 1.000 | 2.000 | 2.000 | 0.000 | -2.000 | 1.000 | -4.289 |
| 153 | 457 | ATC | 3 | 0 | 0.814 | 2.186 | 3.685 | 0.000 | -3.685 | 1.000 | -7.903 |
| 154 | 460 | CTG | 4 | 0 | 1.789 | 1.208 | 2.236 | 0.000 | -2.236 | 1.000 | -4.795 |
| 155 | 463 | CAC | 1 | 0 | 0.791 | 2.209 | 1.263 | 0.000 | -1.263 | 1.000 | -2.710 |
| 156 | 466 | CTT | 6 | 0 | 1.685 | 1.306 | 3.562 | 0.000 | -3.562 | 1.000 | -7.639 |
| 157 | 469 | CTC | 0 | 0 | 1.000 | 2.000 | 0.000 | 0.000 | 0.000 | N/A | 0.000 |
| 158 | 472 | TTT | 0 | 0 | 0.880 | 2.120 | 0.000 | 0.000 | 0.000 | N/A | 0.000 |
| 159 | 475 | TTA | 1 | 0 | 1.735 | 1.195 | 0.576 | 0.000 | -0.576 | 1.000 | -1.236 |
| 160 | 478 | CAC | 0 | 0 | 0.790 | 2.210 | 0.000 | 0.000 | 0.000 | N/A | 0.000 |
| 161 | 481 | GAA | 1 | 0 | 0.853 | 2.115 | 1.172 | 0.000 | -1.172 | 1.000 | -2.513 |
| 162 | 484 | ACG | 2 | 0 | 1.000 | 1.918 | 2.000 | 0.000 | -2.000 | 1.000 | -4.289 |
| 163 | 487 | GGA | 3 | 0 | 1.000 | 1.205 | 3.000 | 0.000 | -3.000 | 1.000 | -6.434 |
| 164 | 490 | TCA | 1 | 0 | 1.000 | 1.871 | 1.000 | 0.000 | -1.000 | 1.000 | -2.145 |
| 165 | 493 | AAC | 0 | 0 | 0.790 | 2.210 | 0.000 | 0.000 | 0.000 | N/A | 0.000 |
| 166 | 496 | AAC | 0 | 0 | 0.790 | 2.210 | 0.000 | 0.000 | 0.000 | N/A | 0.000 |
| 167 | 499 | CCG | 1 | 0 | 1.000 | 2.000 | 1.000 | 0.000 | -1.000 | 1.000 | -2.145 |
| 168 | 502 | GCC | 0 | 0 | 1.000 | 2.000 | 0.000 | 0.000 | 0.000 | N/A | 0.000 |
| 169 | 505 | GGA | 1 | 0 | 1.000 | 1.102 | 1.000 | 0.000 | -1.000 | 1.000 | -2.145 |
| 170 | 508 | TTA | 5 | 0 | 1.771 | 1.160 | 2.823 | 0.000 | -2.823 | 1.000 | -6.055 |
| 171 | 511 | AAT | 1 | 0 | 0.870 | 2.130 | 1.149 | 0.000 | -1.149 | 1.000 | -2.465 |
| 172 | 514 | TCT | 0 | 0 | 1.000 | 2.000 | 0.000 | 0.000 | 0.000 | N/A | 0.000 |
| 173 | 517 | GAT | 1 | 1 | 0.854 | 2.146 | 1.171 | 0.466 | -0.705 | 0.919 | -1.511 |
| 174 | 520 | GCA | 5 | 0 | 1.000 | 2.000 | 5.000 | 0.000 | -5.000 | 1.000 | -10.723 |
| 175 | 523 | GAT | 0 | 0 | 0.880 | 2.120 | 0.000 | 0.000 | 0.000 | N/A | 0.000 |
| 176 | 526 | AAA | 1 | 0 | 0.848 | 1.248 | 1.180 | 0.000 | -1.180 | 1.000 | -2.530 |
| 177 | 529 | ATT | 1 | 1 | 0.870 | 2.130 | 1.149 | 0.470 | -0.680 | 0.916 | -1.457 |
| 178 | 532 | TCT | 0 | 0 | 1.000 | 2.000 | 0.000 | 0.000 | 0.000 | N/A | 0.000 |
| 179 | 535 | TTC | 0 | 0 | 0.790 | 2.210 | 0.000 | 0.000 | 0.000 | N/A | 0.000 |
| 180 | 538 | CAC | 0 | 0 | 0.790 | 2.210 | 0.000 | 0.000 | 0.000 | N/A | 0.000 |

Abbreviations in title: Syn, synonymous; Nonsyn, nonsynonymous; s: the number of synonymous substitutions; n, the number of nonsynonymous substitutions; S: synonymous sites; N, nonsynonymous sites; dS, the number of synonymous substitutions per site (s/S); dN, the number of nonsynonymous substitutions per site (n/N); N/A, not available.
